# A new obligate CXCL4–CXCL12 heterodimer for studying chemokine heterodimer activities and mechanisms

**DOI:** 10.1038/s41598-022-21651-0

**Published:** 2022-10-13

**Authors:** Khanh T.P. Nguyen, Brian Volkman, Didier Dréau, Irina V. Nesmelova

**Affiliations:** 1 Department of Biological Sciences, University of North Carolina, Charlotte, NC USA; 2 Department of Biochemistry, Medical College of Wisconsin, Milwaukee, WI USA; 3 Department of Physics and Optical Sciences, University of North Carolina, Charlotte, NC USA; 4 School of Data Science, University of North Carolina, Charlotte, NC USA

**Keywords:** Biophysics, Chemical biology, Structural biology

## Abstract

Chemokines form a family of proteins with critical roles in many biological processes in health and disease conditions, including cardiovascular, autoimmune diseases, infections, and cancer. Many chemokines engage in heterophilic interactions to form heterodimers, leading to synergistic activity enhancement or reduction dependent on the nature of heterodimer-forming chemokines. In mixtures, different chemokine species with diverse activities coexist in dynamic equilibrium, leading to the observation of their combined response in biological assays. To overcome this problem, we produced a non-dissociating CXCL4–CXCL12 chemokine heterodimer OHD_4–12_ as a new tool for studying the biological activities and mechanisms of chemokine heterodimers in biological environments. Using the OHD_4–12_, we show that the CXCL4–CXCL12 chemokine heterodimer inhibits the CXCL12-driven migration of triple-negative MDA-MB-231 breast cancer cells. We also show that the CXCL4–CXCL12 chemokine heterodimer binds and activates the CXCR4 receptor.

## Introduction

Chemokine signaling is essential in normal physiologic and pathological conditions^1,2^. Chemokine signaling is mediated by intermolecular interactions with G protein-coupled receptors (GPCRs), cell surface glycosaminoglycans (GAGs), and through chemokine homooligomerization^3,4^. Additionally, different chemokines can interact with each other to form heterodimers. These heterophilic interactions have been directly detected by several methods, including co-immunoprecipitation and ligand blot^5–12^, surface plasmon resonance^9,11^, mass spectrometry^8,13^, and NMR (Nuclear Magnetic Resonance) spectroscopy^9,12–17^. A pairwise bidirectional immunoblot chemokine screening shows that heterophilic interactions are abundant in the chemokine family identifying approximately 200 distinct interactions^12^.

The role of heterophilic interactions in chemokine signaling remains to be understood. However, it is established that cell responses to chemokine mixtures differ from individual chemokines^18–24^. Treatment with chemokine mixtures demonstrated either a synergistically enhanced or a reduced activity dependent on the microenvironment and the nature of heterodimer-forming chemokines tested^6,7,11,12,15,16,19,22,23,25^. For example, the heterodimerization of platelet-derived CXCL4 with CXCL8 chemokine inhibits the activation and proliferation of endothelial cells, and the CXCL8-induced migration of cells transfected with the CXCR2 chemokine receptor^14,15^, whereas CXCL4-CCL5 chemokine heterodimerization promotes the arrest of CCL5-stimulated monocytes on activated endothelium^11^. In addition, chemokines CXCL10 and CCL22, co-expressed in the inflamed skin, synergistically enhance the CCR4-mediated chemotaxis of T cells^25^. Likewise, CCR7 chemokine ligands CCL19 and CCL21 enhance monocytes recruitment by forming heterodimers with CCL7 and CCL2^22^, while heterodimers formed by CXCL9 and CXCL12 chemokines co-expressed in the perivascular area of the tumor enhance CXCR4-mediated recruitment of tumor-infiltrating lymphocytes and malignant B cells^6^.

The in vivo co-localization, particularly near GAGs, produces favorable conditions for chemokine heterodimerization. In fact, a few studies have already demonstrated the relevance of the heterophilic interactions in in vivo animal models and the possibility of targeting such interactions for therapeutic benefit^9,12,26^. For example, the disruption of the CCL5-CXCL4 heterodimer decreased the CCL5-mediated neutrophil influx, edema formation, and destruction of lung tissue in acute lung injury^26^ and attenuated monocyte recruitment, thereby reducing atherosclerosis in mice^9^. However, the use of heterophilic interactions as drug targets requires an understanding of the mechanism of action of chemokine heterodimers, which is currently lacking.

In chemokine mixtures, competing homophilic and heterophilic interactions lead to the equilibrium coexistence of different chemokine species including monomers, dimers, heterodimers, and, in some cases, higher-order oligomers (reviewed in^27,28^). The measured biological outcomes represent the combined response to chemokine species, rendering the direct functional assessment of the heterodimer biological activity challenging. Here, we overcome this limitation by generating a new, non-dissociating heterodimer of CXCL4 (platelet factor 4) and CXCL12 (stromal cell-derived factor-1) chemokines, OHD_4–12_ (Obligate HeteroDimer CXCL4–CXCL12). CXCL4 and CXCL12 are both stored and released upon stimulation of platelets^29^, thus having ample opportunities to interact in vivo and modulate cell responses in chemokine-rich microenvironments. We also provide proof-of-principle evidence that OHD_4–12_ is a valuable tool for investigating the biological activities and the mechanism of action of the CXCL4–CXCL12 chemokine heterodimer. In particular, we show that the OHD_4–12_ binds and activates the CXCL12 receptor CXCR4 and inhibits the CXCL12-driven migration of MDA-MB-231 breast cancer cells. The mechanistic insight obtained from using the OHD_4–12_ may extend to other chemokine heterodimers and inform experiments testing their mode of action.

## Results

### Design and production of the obligate CXCL4–CXCL12 heterodimer OHD_4–12_

**Figure 1:**
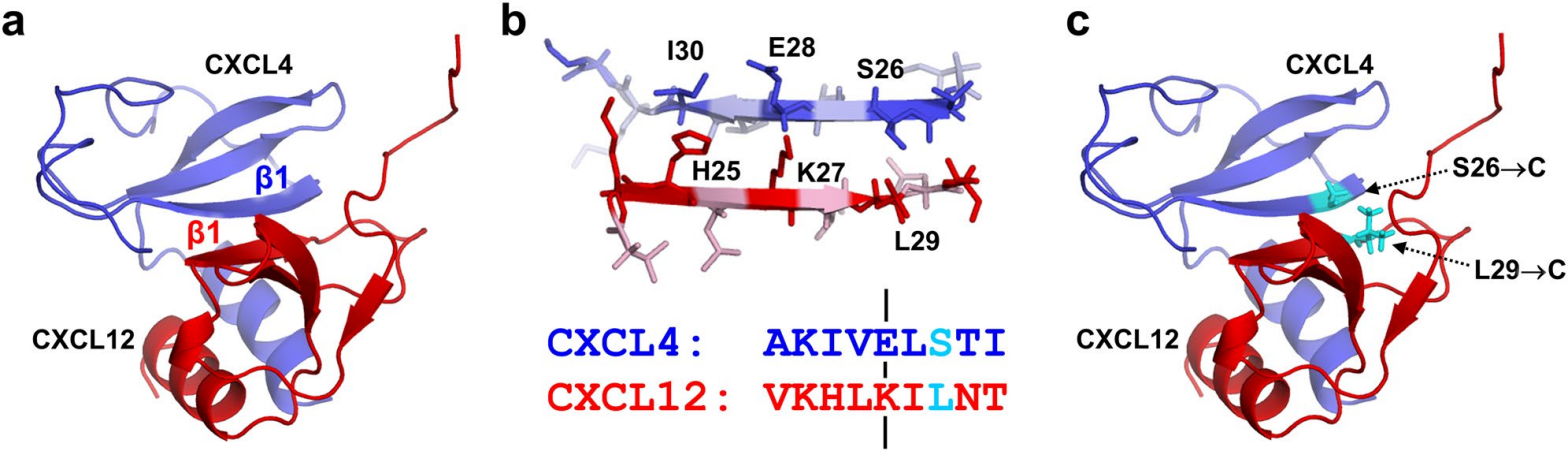
(**a**) Structural model of the CXCL4–CXCL12 heterodimer. The CXCL4 monomer is shown in blue and the CXCL12 monomer is shown in red. First beta-strands forming the intermonomer interface are labeled. (**b**) The structure (top) and the amino acid sequences (bottom) of first beta-strands from CXCL4 (blue) and CXCL12 (red), representing the intermonomer interface, where several amino acids with side chains on the same side of beta-strands are labeled. The axis of symmetry is indicated by black lines on the amino acid sequences of shown beta-strands, and residues selected for mutation are colored in cyan. (**c**) Structural model of the CXCL4–CXCL12 heterodimer with amino acid residues selected for mutation labeled.

Our previous results showed that CXCL4 and CXCL12 chemokines formed heterodimers in in vitro biophysical conditions and in vivo in human platelets^8^. Using experimental NMR titration data and computational modeling^8,16^, we determined that CXCL4 and CXCL12 chemokines formed the heterodimer of CXC-type, in which the first beta-strand β1 from each chemokine monomer participated in the inter-monomer interface (Fig. 1a). We used this molecular model to generate an obligate, e.g., a non-dissociating CXCL4–CXCL12 heterodimer, termed OHD_4–12_. To generate the OHD_4–12_, we utilized the disulfide trapping strategy^17,30,31^, because the inter-monomer interface of the CXC-type CXCL4–CXCL12 heterodimer is suitable for introducing cysteine amino acid residues for disulfide bond formation.

The rationale for amino acid residue selection for cysteine substitutions was as follows. First, CXCL4 and CXCL12 chemokines can form the CXC-type homodimers. Therefore, to avoid the formation of disulfide-linked homodimers, we excluded from consideration residues that directly face each other at the homodimer interface, i.e., E28 in CXCL4 and K27 in CXCL12 (Fig. 1b). For the same reason, we also excluded from consideration residues located next to E28 and K27, i.e., residues in positions 27 and 29 in CXCL4 and 26 and 28 in CXCL12. Next, we considered two pairs of residues located one residue apart from E28 and K27 and having side chains pointing in the same direction to facilitate the disulfide bond formation when substituted for cysteines. These residue pairs were S26-L29 and I30-H25 (the first listed residue is from CXCL4 and the second residue is from CXCL12). While these residues were still located at the heterodimer interface, they were sufficiently far from the symmetry axis to form disulfide-bonded homodimers. Finally, while either pair was suitable for cysteine substitution, we selected S26 in CXCL4 and L29 in CXCL12 (Fig. 1b) because at least serine-to-cysteine substitution was conservative and did not alter the charge state of the intermonomer interface.

**Figure 2:**
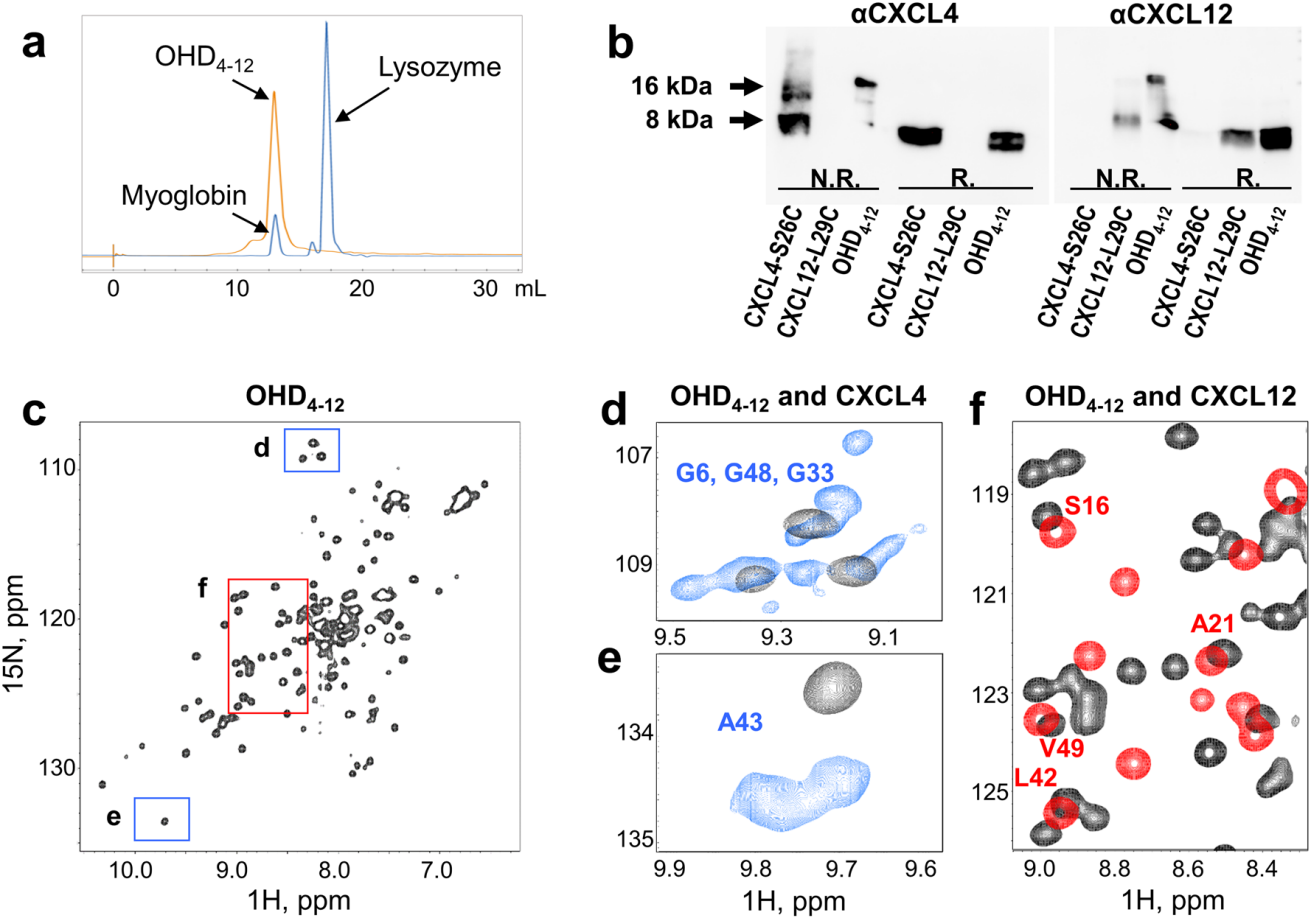
(**a**) Size-exclusion chromatograms. The elution profiles of lysozyme (14.4 kDa) and myoglobin (17 kDa) in blue and OHD_4–12_ (15.9 kDa) in orange are shown. (**b**) WB analysis in non-reduced (left bands, N.R.) and reduced (right bands, R.) conditions demonstrates the presence of OHD_4–12_ obtained following the mixing of CXCL4-S26C and CXCL12-L29C mutants in the presence of Cu^2+^ detected with anti-CXCL4 (αCXCL4) or anti-CXCL12 (αCXCL12) antibodies (see also Supplementary Fig. S1). (**c**) NMR spectroscopic analysis of OHD_4–12_ folding state. The 15N-HSQC NMR spectrum of the uniformly 15N-labeled 68 μM OHD_4–12_ in 90% H2O/10% D_2_O at pH 6.9 in the presence of 20 mM NaCl, collected at 40 °C. (**d**–**f**) Expansions from the OHD_4–12_ spectrum overlaid with CXCL4_wt_ (blue) and CXCL12_wt_ (red) 15N-HSQC NMR spectra. Known signal assignments for CXCL4 and CXCL12 are indicated. The 15N-HSQC NMR spectrum of the uniformly 15N-labeled 150 μM CXCL4 in 90% H2O/10% D_2_O at pH 5.0 in the presence of 20 mM NaCl was collected at 40 °C. The 15N-HSQC NMR spectrum of the uniformly 15N-labeled 51 μM CXCL12 in 20 MES buffer prepared with 90% H2O/10% D_2_O at pH 6.8 was collected at 25 °C. The difference in experimental conditions was due to the difference in solubility properties of CXCL4 and CXCL12.

CXCL4-S26C and CXCL12-L29C mutants were expressed and purified individually as detailed in the Methods section. To form the heterodimer, we mixed the mutants at a 1:1 molar ratio in phosphate buffer containing a catalytic amount of Cu^2+^ as an oxidizing agent^32^. After the incubation for 18 h at 4 °C, the OHD_4–12_ was purified from the reaction mixture by size-exclusion chromatography. The elution time of the 15.7 kDa OHD_4–12_ was approximately the same as of the 17 kDa myoglobin and smaller than of the 14.6 kDa lysozyme (Fig. 2a), indicating that the produced species had the correct molecular weight. Additionally, the Western Blot (WB) analysis in non-reducing (bands on the left) and reducing (bands on the right) conditions further verified the formation of the OHD_4–12_ with an expected molecular weight of ~ 16 kDa (Fig. 2b, the full gel is shown in Supplementary Fig. S1). Finally, the formation of the OHD_4–12_ was confirmed in co-immunoprecipitation (co-IP) experiments using antibodies specific to CXCL4 and CXCL12. The CXCL4-S26C or CXCL12-L29C fractions were first immunoprecipitated with magnetic microbeads coated with anti-CXCL4 or anti-CXCL12 antibodies and then detected using anti-CXCL12 antibodies, demonstrating the presence of the OHD_4–12_ heterodimer (Supplementary Fig. S2).

We then used NMR spectroscopy to assess the folding state of the OHD_4–12_. The CXCL4-S26C and CXCL12-L29C mutants were individually uniformly 15N-enriched and used to produce the uniformly 15N-enriched OHD_4–12_. The 15N-HSQC (heteronuclear single-quantum coherence) NMR spectrum of the 15N-OHD_4–12_ displayed well-dispersed cross-peaks, which confirmed the presence of a folded structure (Fig. 2c). The number of cross-peaks corresponded to the number of amino acids in the OHD_4–12_, indicating the presence of a single heterodimer species. This simplified pattern contrasts the 15N-HSQC spectrum of 15N-enriched CXCL4, for which multiple resonances represent most amino acids due to the intermediate-to-slow exchange equilibrium between CXCL4 monomers, homodimers, and homotetramers on the NMR time scale and the asymmetry of CXCL4 homotetramer^15,33,34^. Figure 2d,e show representative expansions of overlaid 15N-HSQC spectra of 15N-CXCL4 (blue cross-peaks) and 15N-OHD_4–12_ (black cross-peaks). Unlike CXCL4, the CXCL12 chemokine produces the 15N-HSQC NMR spectrum, in which a single resonance represents each amino acid. Some heterodimer cross-peaks originating from the CXCL12 counterpart can be tracked by comparing the 15N-HSQC NMR spectra of the 15N-OHD_4–12_ (black cross-peaks) and 15N-CXCL12 (red cross-peaks). Some of these peaks are labeled in Fig. 2f, showing an expansion of the overlaid spectra of the OHD_4–12_ and CXCL12. Supplementary Fig. S3 provides the overlay of full 15N-HSQC NMR spectra of the OHD_4–12_, CXCL12, and CXCL4 with known assignments for CXCL4 and CXCL12 labeled.

### OHD_4–12_ inhibits the CXCL12-induced migration of breast cancer MDA-MB-231 cells

**Figure 3:**
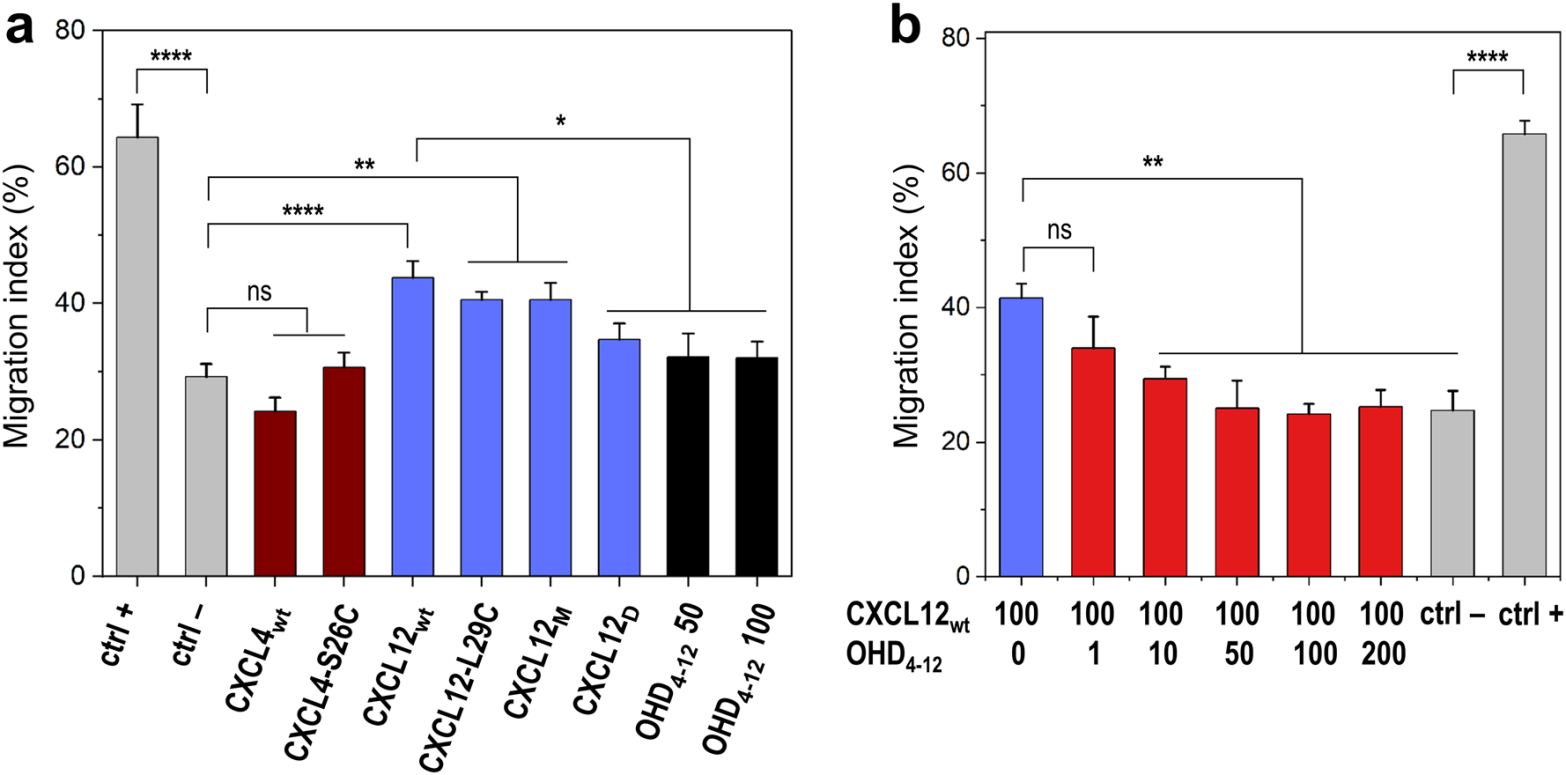
MDA-MB 231 breast cancer cells migration. (**a**) Migration of MDA-MB 231 cells treated with 100 nM of CXCL4_wt_, its mutant CXCL4-S26C used as a CXCL4 counterpart to produce OHD_4–12_, CXCL12_wt_, its mutant CXCL12-L29C used as a CXCL12 counterpart to produce OHD_4–12_, CXCL12 variants CXCL12_M_ (obligate monomer) and CXCL12_D_ (obligate dimer), and 50 or 100 nM of OHD_4–12_. Negative and positive controls were 0 and 10% FBS. Migration index was determined as a percentage of wound healing in the absence of chemokine treatment at 0% FSB. (**b**) Competitive inhibition of CXCL12-driven migration of MDA-MB 231 cells by OHD_4–12_ at concentrations ranging from 1 to 200 nM. The OHD_4–12_ inhibits CXCL12-induced migration of MDA-MB 231 cells in a dose–response manner. The migration of cells treated with 100 nM CXCL12_wt_ alone is shown for comparison. Negative and positive controls were as in panel (**a**). All presented data are means ± SEM (standard errors of the means) from n ≥ 3 independent experiments. *p < 0.05; **p < 0.01; ****p < 0.0001; analyzed by one-way ANOVA followed by a post-hoc Tukey multiple comparison test.

Next, we sought to determine whether the CXCL4–CXCL12 chemokine heterodimer is a biologically active species. Previously, we demonstrated that the addition of CXCL4 to CXCL12 chemokine led to the inhibition of CXCL12-induced migration of triple-negative MDA-MB-231 breast cancer cells in a wound-healing assay^16^. Here, we similarly assessed the effect of the OHD_4–12_ on the migration of MDA-MB-231 cells. As expected, the wild-type CXCL12 (CXCL12_wt_) and the mutant CXCL12-L29C, used to produce the disulfide-trapped heterodimer, induced the migration of MDA-MB-231 cells (Fig. 3a). We also observed the differential effect of the obligate CXCL12 monomer (CXCL12_M_) and dimer (CXCL12_D_) on cell migration^35^ (Fig. 3a). Cell response to CXCL12_M_ was comparable to the wild-type CXCL12_wt_ and CXCL12-L29C, whereas CXCL12_D_ had a lesser effect. In contrast to CXCL12, at the concentration used, the wild-type CXCL4_wt_ and its mutant CXCL4-S26C, used to produce the disulfide-trapped heterodimer, did not significantly affect the MDA-MB-231 cell migration (Fig. 3a).

Several possible activities of the CXCL4–CXCL12 heterodimer, constructed from two chemokines with opposite effects on cell migration, can be expected: no effect or inhibition of cell migration as with CXCL4_wt_ or CXCL4-S26C, notably or weakly enhanced cell migration as with CXCL12_wt_, CXCL12-L29C, CXCL12_M_ or CXCL12_D_, and new activity^12,36^. Our data show that the OHD_4–12_ alone has no significant effect on MDA-MB-231 cell migration at concentrations of 50 and 100 nM (Fig. 3a). However, when added to CXCL12, the OHD_4–12_ inhibits the CXCL12-driven MDA-MB-231 cell migration at a concentration equivalent to 2:1, 1:1, and 1:2 (CXCL12:heterodimer) molar ratios (Fig. 3b).

### Involvement of chemokine receptors CXCR4 and CXCR3 in the OHD_4–12_ activity

Two chemokine counterparts of the OHD_4–12_, CXCL12, and CXCL4, bind receptors CXCR4 and CXCR7 (ACKR3) or CXCR3b, respectively^37–41^. Previously, we determined that MDA-MB-231 breast cancer cells strongly express CXCR4 and CXCR3b receptors^16^. The expression of the CXCR7 receptor on MDA-MB-231 cells is marginally low^42,43^. In our experimental setting, the percent of CXCR7 receptor expression on MDA-MB-231 cells was 1.6 ± 0.1, whereas the CXCR4 and CXCR3b expression was 23.5 ± 2.7 and 38.0 ± 0.2%, respectively (Supplementary Fig. S4). Accordingly, MDA-MB-231 breast cancer cells present a uniquely suitable model system for investigating the involvement of CXCR4 and CXCR3 receptors in the CXCL4–CXCL12 heterodimer signaling. Indeed, low expression of the CXCR7 receptor allows the assessment of the OHD_4–12_ signaling through the CXCR4 receptor without CXCR7 interference, either as a CXCL12 scavenger^44,45^ or through CXCR4-CXCR7 heterodimerization^46^.

**Figure 4:**
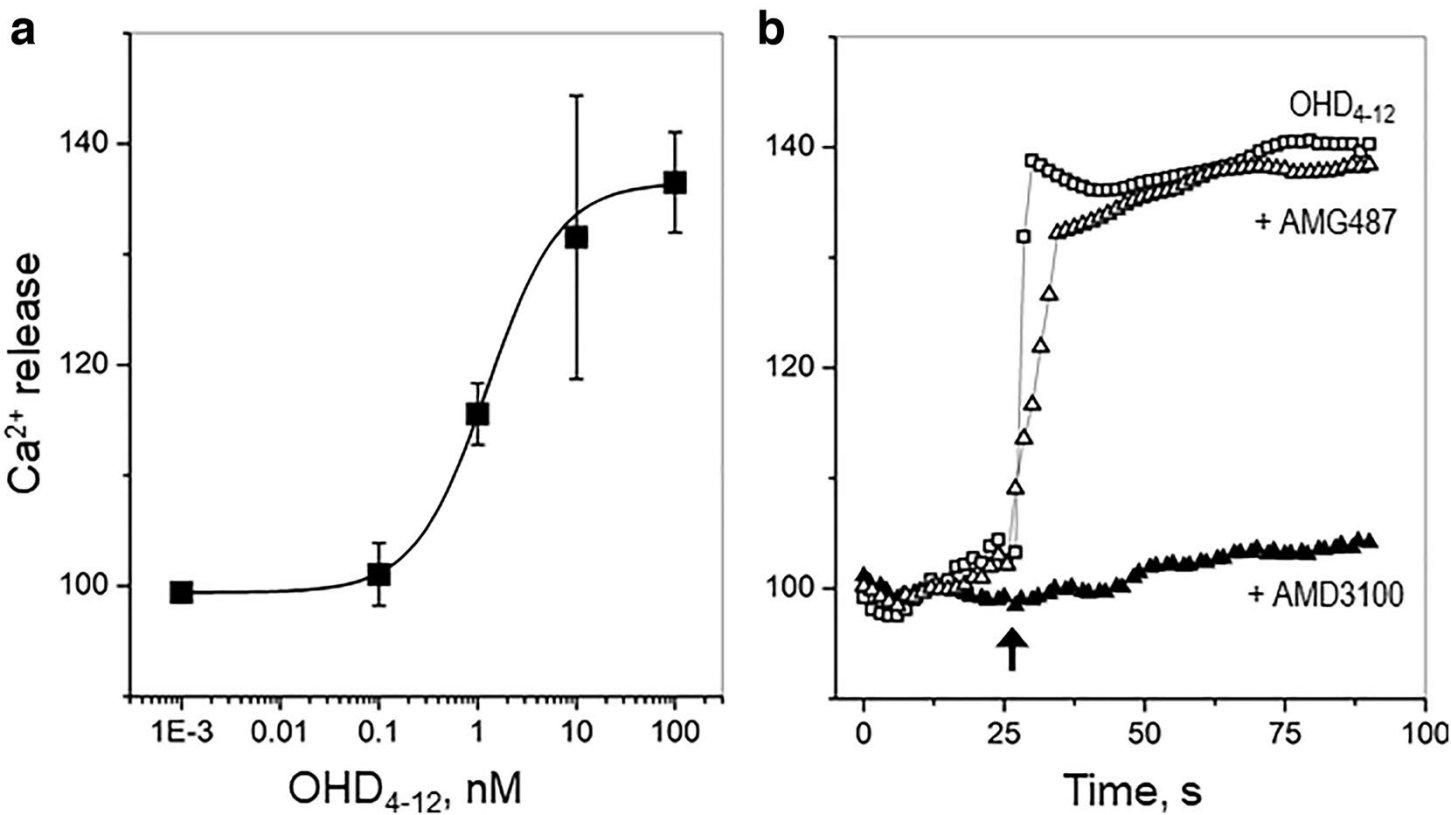
The cytoplasmic Ca^2+^ release in MDA-MB-231 breast cancer cells induced by OHD_4–12_. (**a**) The dose–response curve of intracellular Ca^2+^ release induced by OHD_4–12_. Solid line represents the best fit of experimental data using the logistic function. (**b**) The cytoplasmic Ca^2+^ release in MDA-MB-231 cells induced by 100 nM OHD_4–12_ (open squares). The arrow indicates the time-point of chemokine addition. The pre-incubation of cells with the CXCR4 antagonist AMD3100 (20 nM, solid triangles) inhibited, whereas the pre-incubation of cells with the CXCR3 antagonist AMG487 (5 nM, open triangles) had no effect on the OHD_4–12_-induced intracellular Ca^2+^ release.

In highly invasive cells, such as MDA-MB-231 breast cancer cells, the binding of CXCL12 to CXCR4 activates multiple downstream signaling pathways, including calcium mobilization^47^. First, we verified that CXCL12_wt_, CXCL12-L29C, CXCL12M, and CXCL12D, but not CXCL4-S26C or CXCL12_wt_ mixed with the specific CXCR4 inhibitor AMD3100 led to the increase of cytoplasmic Ca^2+^ in MDA-MB-231 breast cancer cells (Supplementary Fig. S5). Then, we tested the ability of the OHD_4–12_ to bind and activate the CXCR4 receptor by monitoring changes in cytoplasmic calcium (Ca^2+^ release) incubated with different amounts of the OHD_4–12_. The addition of OHD_4–12_ to MDA-MB-231 cells induced a dose-dependent increase of cytoplasmic Ca^2+^ (Fig. 4a) with the half-maximal effective concentration (EC50) of 1.3 ± 0.1 nM. The addition of the specific CXCR4 inhibitor AMD3100 abrogated the Ca^2+^ release (Fig. 4b), confirming that the OHD_4–12_ activated the downstream signaling of the CXCR4 receptor. In contrast, the addition of the specific CXCR3 inhibitor AMG487 had no effect (Fig. 4b), demonstrating that the OHD_4–12_ does not activate those CXCR3 signaling pathways that lead to calcium mobilization at least at concentrations up to 100 nM.

## Discussion

The formation of heterodimers by different chemokines is established experimentally, in in vitro and in vivo settings^6–9,11–13,15–17,19,22,23,25,31^. However, whether these heterodimers are biologically active species with unique functions or the response to chemokine mixtures is simply a combination of cell responses to individual chemokines remains debated and the mode of action of chemokine heterodimers remains to be determined. In this work, our primary goal was to determine whether the CXCL4–CXCL12 heterodimer possesses its own biological activity and, for the mechanistic insight, whether it can bind and activate receptors of chemokines forming the heterodimer.

The existence of the equilibrium of interconverting chemokines species in situ (i.e., reviewed in^28,48,49^) does not permit the investigation of cell response to chemokine heterodimers apart from chemokine monomers, homodimers, and, possibly, higher-order oligomers. Therefore, a powerful strategy to address this challenge is to use obligate, non-dissociating chemokine heterodimers in functional studies^12,17,31,36^. Here, we generated a new, obligate CXCL4–CXCL12 chemokine heterodimer, named OHD_4–12_. The CXC-type arrangement of chemokine monomers forming the CXCL4–CXCL12 heterodimer^8,16^ is particularly suitable for introducing cysteine substitutions at the intermonomer interface for disulfide bond formation. Therefore, we followed the disulfide-trapping strategy^17,30,31^ for forming the OHD_4–12_.

The CXCL4–CXCL12 heterodimer-forming chemokine pair was selected because of the high probability of forming heterodimers in vivo. Both chemokines are stored in platelets and released after platelet activation^29^. Indeed, previously we showed the physical interaction of CXCL4 and CXCL12 chemokines by co-immunoprecipitating them from human platelets^8^. Furthermore, the interaction with GAGs facilitates the accumulation and localization of chemokines in situ to establish gradients (e.g., reviewed in^4^), including CXCL12 and CXCL4. The GAG-binding of a few chemokine heterodimers was characterized^17,31^ and it was also shown that binding to GAGs stabilized chemokine heterodimers^13^. Thus, it is plausible that the two heterodimer-forming chemokines dissociate from GAGs as a heterodimer, or remain close to each other to form a heterodimer, readily available for receptor binding. Further interest in CXCL4–CXCL12 heterodimer comes from the critical role of the CXCL12 chemokine and its receptor, CXCR4, in invasion, migration, and proliferation steps of tumor progression in more than 75% of all cancers, including breast, ovarian, lung, colon, prostate, kidney, melanoma, brain, esophageal, pancreatic, and various forms of leukemia^50,51^. Indeed, organs with the highest levels of CXCL12 expression (lymph nodes, lung, liver, and bone marrow) are the most common sites of metastasis for breast cancer cells^51,52^. CXCL4 is abundant in platelets of healthy individuals. The levels of CXCL4 and CXCL12 can be further elevated in cancer patients^53,54^ leading to micromolar concentrations at the tumor or metastasis site that could favor CXCL4–CXCL12 heterodimerization in vivo. Therefore, we tested the activity of the OHD_4–12_ on the migration of invasive MDA-MB-231 breast cancer cells. MDA-MB-231 are triple negative breast cancer cells extensively used in the investigation of therapeutic targets as well as mechanistic studies of cancer cell migration^55–57^. Although other cells, including different types of cancer cells, may trigger stronger than MDA-MB-231 cell migration responses^58^, MDA-MB-231 cells uniquely express CXCR4 and CXCR3b but not CXCR7 (^16,42,43^ and Supplementary Fig. S4). This feature makes MDA-MD-231 uniquely suitable for the investigation of CXCL12-CXCL4 heterodimer signaling.

Previously, we demonstrated that the addition of CXCL4 inhibited the CXCL12-induced migration of MDA-MB-231 cells in a dose-dependent manner and that a CXCL4-derived peptide mimicking the CXCL4–CXCL12 inter-monomer interface retained the inhibitory activity of CXCL4, suggesting that the CXCL4–CXCL12 heterodimer was at least partially responsible for the observed effect^16^. In the current study, by using the OHD_4–12_, we explicitly proved that the CXCL4–CXCL12 heterodimer was an active species that inhibited the migration of MDA-MB-231 breast cancer cells driven by the CXCL12 chemokine and established the role of CXCL4–CXCL12 heterodimer in MDA-MB-231 breast cancer cell migration. In addition, our data support the paradigm that chemokine activity can be inhibited by CXC-type heterodimers^12^.

Chemokines exert their functions by binding and activating GPCRs. In a generally applicable to all chemokines basic two-site model, chemokine signaling starts with the formation of an extensive protein-receptor interface between the unstructured N terminus of the receptor (chemokine recognition site 1—CRS1) and the globular core, N loop, and 40 s loop of the chemokine^59^. Subsequently, the flexible N-terminus of the chemokine interacts with other extracellular receptor residues and docks into a pocket within the transmembrane domain of the receptor (chemokine recognition site 2—CRS2), inducing conformational changes that lead to signaling^59–61^. The formation of the CXC-type CXCL4–CXCL12 heterodimer does not involve the N-terminus, N loop, or the 40 s loop of the chemokine ligand, and they remain available for interaction with the receptor (Fig. 1a)^16^. Our data show that the OHD_4–12_ can bind and activate the CXCL12’s receptor CXCR4 with the EC50 of 1.3 ± 0.1 nM. The value of EC50, measured for Ca^2+^ release induced by the OHD_4–12_, is comparable to EC50 values reported in the literature^30,62,63^ or this work (1.0 ± 0.5 nM, Supplementary Fig. S5) for the wild-type CXCL12.

The observation that the OHD_4–12_ binds and activates the CXCR4 receptor is in accordance with previous observations that disulfide-trapped CXCL1-CXCL7 heterodimer binds and activates the CXCR2 receptor^17^, whereas CC-type obligate CCL5-CCL17 heterodimer involves both corresponding receptors CCR4 and CCR5^12^. These observations suggest that chemokine heterodimers may act by involving receptors of both heterodimer-forming chemokines. Our data show that the addition of the specific CXCR4 inhibitor AMD3100 abrogates the OHD_4–12_-induced Ca^2+^ release, whereas the addition of the specific CXCR3 inhibitor AMG487 does not (Fig. 4b), indicating that CXCR3 signaling pathways leading to calcium mobilization are not activated by 100 nM OHD_4–12_. However, these data do not entirely exclude the involvement of the CXCR3 receptor. First, OHD_4–12_ may activate CXCR3 signaling pathways not tested in this work, such as activation of adenylyl cyclase activity^41^. Second, the lack of CXCR3-mediated Ca^2+^ release observed in this work may be due to significantly higher concentrations of OHD_4-12_ (i.e., its CXCL4 counterpart) required for the activation of CXCL4-CXCR3 Ca^2+^ signaling. Indeed, Korniejewska et al.^64^ used 10 μM CXCL4, and Mueller et al.^65^ reported that 500 nM CXCL4 was required to induce intracellular calcium flux in activated T cells. The 20-fold concentration discrepancy was proposed to be related to the difference in stimuli used for cell activation^64^. Further investigation will reveal whether the OHD_4–12_ can bind and activate the CXCR3 receptor.

The difference in effects of CXCL12_M_ (e.g., the CXCL12-H25R mutant) and CXCL12_D_ (disulfide-linked) on the migration of colon cancer HCT116 and HT29 and monocytic leukemia THP-1 cells was previously observed^30,35^. CXCL12_D_ competitively blocked CXCL12_wt_-induced cell migration and, in contrast to CXCL12_M_, was not able to stimulate the migration of these cells at the concentration of 10 nM^35^. Our data parallel this result as we observe a reduced migration of MDA-MB-231 cells treated with 100 nM CXCL12_D_ as compared to CXCL12_wt_ or CXCL12_M_ and CXCL12-L29C variants. The difference in CXCL12_M_ and CXCL12_D_ activity stems from different binding modes to the CXCR4 receptor^30,35,62^. In the case of CXCL12_M_, the N-terminal domain of the CXCR4 receptor wraps around the chemokine and forms a beta-sheet with its first beta-strand β1, leading to an active signaling complex that promotes chemotaxis^35,62,66^. The lack of the CXCL12_D_ chemotactic activity is caused by the inaccessibility of the β1 strand of the CXCL12 monomer for the interaction with the receptor because it participates in the inter-monomer contact with the β1 strand of an opposing CXCL12 monomer^30,62^.

Similar to CXCL12_D_, we observe no significant effect of the OHD_4–12_ on the migration of MDA-MB-231 breast cancer cells and the inhibition of the CXCL12_wt_-induced MDA-MB-231 cell migration by the OHD_4–12_. The design of the OHD_4–12_ (Fig. 1) requires that the β1 strand of the CXCL12 monomer in the CXCL4–CXCL12 heterodimer is involved in the inter-monomer interface with the CXCL4 monomer and has to be similarly inaccessible for the interactions with the CXCR4 receptor as in CXCL12_D_. Thus, both the CXCL12_D_ and the OHD_4–12_ may have a similar mode of receptor activation leading to cell migration. This observation may further extend to the CXCL4–CXCL12 heterodimers formed in vivo.

In summary, we present a new obligate heterodimer OHD_4–12_, a tool for investigating the CXCL4–CXCL12 heterodimer functionalities in vitro or in vivo. Using the OHD_4–12_, we demonstrate that it interrupts the CXCL12-driven migration of breast cancer cells, thus establishing the role of CXCL4–CXCL12 heterodimer in breast cancer and suggesting its utility for therapeutic advantage. Furthermore, our results on CXCR4 receptor activation by the OHD_4–12_ provide the basis for further mechanistic studies of chemokine heterodimers and are likely to be broadly applicable to chemokine heterodimer-receptor interactions.

## Methods

### Protein expression and purification

Plasmids with DNA encoding CXCL4_wt_, CXCL12_wt_ or CXCL4-S26C and CXCL12-L29C mutants inserted into pET-24d(+) vector (Novagen) were purchased from Genscript. Unlabeled or uniformly 15N-enriched proteins were expressed in BL21(DE3) pLysS E. coli bacteria (Novagen) grown in LB or M9 media, respectively, at 37 °C with shaking at 250 rpm in the presence of 60 μg/mL kanamycin. M9 medium was supplied with 15N-NH_4_Cl (15N, 99%) (Cambridge Isotope Laboratories) as a sole source of nitrogen. Protein production was induced by the addition of IPTG (isopropyl β-d-1-thiogalactopyranoside) to the final concentration of 0.5 mM when the optical density of bacterial culture at 600 nm (OD_600_) reached 0.6. After the addition of IPTG, bacteria were grown for 4 h at 37 °C and harvested by centrifugation for 30 min at 3000 rpm. Bacterial pellet was resuspended in the lysis buffer (3 ml per gram of pellet), contained 50 mM Tris, 1% Triton, 100 mM PMSF (phenylmethylsulfonyl fluoride), and 0.1% of beta-mercaptoethanol (BME), prepared at pH 8.0. Bacteria were disrupted by sonication (Branson Digital Sonifier) on ice at 40% amplitude for 2 s on/0.5 s off with breaks between cycles to prevent overheating. After sonication, bacteria lysates were centrifuged for 1 h at 20,000 rpm and 4 °C. All proteins expressed as inclusion bodies. Inclusion bodies were homogenized by stirring overnight at 4 °C in the extraction buffer prepared at pH 8.0 (12 ml per gram of pellet with inclusion bodies), containing 50 mM Tris, 8 M Urea, and 0.1% BME. Cell debris were removed by centrifugation at 20,000 rpm and 4 °C for 1 h, and the supernatant containing unfolded soluble proteins was used in subsequent purification steps.

The proteins were initially purified by cation exchange chromatography using the ÄKTA pure 25 M FPLC system and HiTrap SP/FF 16/10 column (Cytiva). CXCL4_wt_, CXCL12_wt_, and CXCL4-S26C proteins were eluted using a 0–100% gradient of elution buffer containing 100 mM Tris, 6 M urea, 2 M NaCl, pH 8.0. Following cation exchange chromatography, the refolding of CXCL4_wt_, CXCL12_wt_, and CXCL4-S26C was performed by 1:50 drop-wise dilution with constant stirring in 100 mM Tris buffer containing 10 mM cysteine and 1 mM cystine at pH 8.0. CXCL12-L29C was refolded on-column by gradually decreasing the concentration of urea from 8 to 1 M using 50 mM Tris buffer containing 10 mM reduced glutathione and 1 mM oxidized glutathione at pH 7.3. Following overnight incubation, the column was washed with 50 mM Tris buffer, pH 7.3, containing 50 mM NaCl. Folded CXCL12-L29C was eluted from the column with a 0–2 M gradient of NaCl. All refolded proteins were further purified by heparin affinity chromatography using HiPrep Heparin FF 16/10 column (Cytiva). The proteins were eluted in the 50 mM Tris buffer at pH 7.3 using a 0–2 M NaCl gradient. The CXCL4_wt_ and CXCL4-S26C, eluting at high NaCl concentration, were sufficiently pure and were ready for subsequent experiments. The purity of CXCL12_wt_ and CXCL12-L29C, eluting at ~ 0.3 M NaCl, was additionally improved by size-exclusion chromatography using HiPrep 26/60 Sepharcryl S-200 HR column (Cytiva) and 50 mM sodium phosphate buffer containing 150 mM sodium chloride at pH 7.0.

To form the disulfide-trapped heterodimer OHD_4–12_, mutants CXCL4-S26C and CXCL12-L29C obtained after the heparin affinity chromatography step, were mixed at 1:1 molar ratio and dialyzed against 50 mM sodium phosphate buffer, containing 150 mM NaCl and 10 μM CuCl_2_ at pH 7.0 for 18 h at 4 °C. Following the dialysis, the mixture was centrifuged to remove the precipitated proteins and the OHD_4–12_ was purified using HiTrap Heparin FF 16/10 column (Cytiva). The OHD_4–12_ eluted at 0.5–0.6 M of NaCl. The purity of OHD_4–12_ was polished by size-exclusion chromatography using a Superdex 75 Increase 10/300 GL column (Cytiva).

Obligate CXCL12 monomer (CXCL12_M_) and dimer (CXCL12_D_) were a gift from Protein Foundry, LLC.

### Western blot and co-immunoprecipitation analyses

Chemokine mixtures or the OHD_4–12_ were electrophoretically separated in non-denaturing or denaturing conditions using 16.5% polyacrylamide gel and then transferred to 0.2 μm nitrocellulose membrane using a semi-transfer apparatus (Biorad). Membranes were blocked in Tris-buffered saline containing 0.1% Tween-20 and 5% non-fat milk at 4 °C and incubated with the primary anti-CXCL4 and anti-CXCL12 antibodies (MAB7952 and MAB310, respectively, R&D systems) overnight. The membranes were then incubated with species-specific horseradish-peroxidase (HRP)-conjugated secondary antibodies (#31450, ThermoFisher). Following the incubation with ECL substrate (BioRad), the presence of specific proteins was determined based on chemiluminescence detected using a ChemiDoc Imaging System (BioRad). For co-IP analysis, chemokine mixtures were incubated with magnetic microbeads pre-coated with mouse anti-CXCL4 or anti-CXCL12 antibodies (R&D Systems) at 4 °C for 2 h. After incubation, microbeads were magnetically bound and washed to remove the unbound fractions. After elution, the Western blot analysis of the immune-precipitated samples was performed using goat anti-CXCL12 monoclonal antibodies (R&D Systems).

### NMR spectroscopy

15N-CXCL4 was prepared at the concentration of 150 μM in 90% H2O/10% D_2_O in the presence of 20 mM of NaCl at pH 5.0. 15N-CXCL12 and 15N-OHD_4–12_ (both counterparts were 15N-labeled) were prepared at concentrations 129 and 68 μM, respectively, in 90% H2O/10% D_2_O in the presence of 20 mM of NaCl at pH 6.9. The two-dimensional 15N-HSQC (heteronuclear single quantum coherence) spectrum of CXCL4_wt_ was recorded on the Bruker Avance 950 MHz spectrometer at David H. Murdock Institute (Kannapolis, NC). 15N-HSQC spectra of CXCL12_wt_ and OHD_4–12_ were recorded on the Bruker Avance III 700 MHz NMR spectrometer equipped with a helium-cooled cryoprobe at METRIC, North Carolina State University. All spectra were recorded at 40 °C.

### Cell migration

MDA-MB 231 breast cancer cells (ATCC) were cultured in DMEM/F12 media (Corning) supplemented with 10% FBS (Atlanta Biologics), l-glutamine, Amphotericin B and Gentamycin (Corning). Following an overnight coating with Collagen type I (12 μg/cm^2^, BD Biosciences) at 37 °C with 5% CO_2_ and > 85% humidity and washes of the unbound Collagen I with sterile PBS, 96-well tissue culture plates (Greiner) were seeded (4 × 10^5^ cells/well) with MDA-MB 231 cell suspension in culture media. Cells were grown to confluence and then incubated overnight with fresh media without FBS (0%). The confluent MDA-MB 231 cell monolayers were then scratched using a sterile pipet tip and the wells washed to remove non-adherent cells. Thereafter, cells were incubated with various chemokines and overlapping microphotographs encompassing the entire area of each scratch/wound were taken at the start of the treatment and following the incubation for 9 h using an IX71 Olympus microscope equipped with a DP70 camera and the associated software (Olympus). Overlapping microphotographs were stitched together, and the area of the wound was determined using ImageJ software (NIH). After normalization to the area measured at time 0, results were expressed as percentage of wound healing. Differences between treatments were tested by ANOVA and individual treatment compared through post-hoc Tukey tests with a priori significance threshold set at p < 0.05.

### Calcium release

MDA-MB 231 breast cancer cells (ATCC) were seeded at 4 × 10^5^ cells in 100 μL per well on 96-well tissue culture plates (Greiner) and grown to confluence. After 48 h, cells were starved in serum-free media for 6 h and then incubated (45 min, 37 °C) with the Ca^2+^ intracellular indicator Fura-2 (2 μM). Cells were washed with phosphate buffered saline (PBS). Following injections of increasing concentrations of chemokines (0–250 nM), variations in cytoplasmic Ca^2+^ were measured every 1.5 s for up to 60 s by detecting the 510 nm fluorescence emission ratio following excitation at 340 nm and 380 nm, respectively, using the ID5 plate fluorescence reader (Molecular Device). Background fluorescence was measured for 30 s prior to the addition of chemokines. Fluorescent signals were subsequently normalized to the average background reading.

## Supplementary Information

Supplementary Figures.

## Data Availability

All data that support the findings of this study are included in the article.

## References

[1] Gerard C, Rollins BJ (2001). Chemokines and disease. Nat. Immunol..

[2] Raman D, Sobolik-Delmaire T, Richmond A (2011). Chemokines in health and disease. Exp. Cell Res..

[3] Allen SJ, Crown SE, Handel TM (2007). Chemokine: Receptor structure, interactions, and antagonism. Annu. Rev. Immunol..

[4] Proudfoot, A. E. I., Johnson, Z., Bonvin, P., & Handel, T. M. Glycosaminoglycan interactions with chemokines add complexity to a complex system. *Pharmaceuticals (Basel)*. **10**, 70-95(2017). 10.3390/ph10030070 PMC5620614 28792472

[5] Guan E, Wang J, Norcross MA (2001). Identification of human macrophage inflammatory proteins 1alpha and 1beta as a native secreted heterodimer. J. Biol. Chem..

[6] Venetz D, Ponzoni M, Schiraldi M, Ferreri AJ, Bertoni F, Doglioni C, Uguccioni M (2010). Perivascular expression of CXCL9 and CXCL12 in primary central nervous system lymphoma: T-cell infiltration and positioning of malignant B cells. Int. J. Cancer.

[7] Paoletti S, Petkovic V, Sebastiani S, Danelon MG, Uguccioni M, Gerber BO (2005). A rich chemokine environment strongly enhances leukocyte migration and activities. Blood.

[8] Carlson J, Baxter SA, Dreau D, Nesmelova IV (2013). The heterodimerization of platelet-derived chemokines. Biochim. Biophys. Acta.

[9] Koenen RR, von Hundelshausen P, Nesmelova IV, Zernecke A, Liehn EA, Sarabi A, Kramp BK, Piccinini AM, Paludan SR, Kowalska MA, Kungl AJ, Hackeng TM, Mayo KH, Weber C (2009). Disrupting functional interactions between platelet chemokines inhibits atherosclerosis in hyperlipidemic mice. Nat. Med..

[10] Giri J, Das R, Nylen E, Chinnadurai R, Galipeau J (2020). CCL2 and CXCL12 derived from mesenchymal stromal cells cooperatively polarize IL-10+ tissue macrophages to mitigate gut injury. Cell Rep..

[11] von Hundelshausen P, Koenen RR, Sack M, Mause SF, Adriaens W, Proudfoot AE, Hackeng TM, Weber C (2005). Heterophilic interactions of platelet factor 4 and RANTES promote monocyte arrest on endothelium. Blood.

[12] von Hundelshausen, P., Agten, S. M., Eckardt, V., Blanchet, X., Schmitt, M. M., Ippel, H., Neideck, C., Bidzhekov, K., Leberzammer, J., Wichapong, K., Faussner, A., Drechsler, M., Grommes, J., van Geffen, J. P., Li, H., Ortega-Gomez, A., Megens, R. T., Naumann, R., Dijkgraaf, I., Nicolaes, G. A., Doring, Y., Soehnlein, O., Lutgens, E., Heemskerk, J. W., Koenen, R. R., Mayo, K. H., Hackeng, T. M., & Weber, C. (2017) Chemokine interactome mapping enables tailored intervention in acute and chronic inflammation. *Sci. Transl. Med*. **9**(384), eaah6650 (2017). 10.1126/scitranslmed.aah6650 28381538

[13] Crown SE, Yu Y, Sweeney MD, Leary JA, Handel TM (2006). Heterodimerization of CCR2 chemokines and regulation by glycosaminoglycan binding. J. Biol. Chem..

[14] Dudek AZ, Nesmelova I, Mayo K, Verfaillie CM, Pitchford S, Slungaard A (2003). Platelet factor 4 promotes adhesion of hematopoietic progenitor cells and binds IL-8: novel mechanisms for modulation of hematopoiesis. Blood.

[15] Nesmelova IV, Sham Y, Dudek AZ, van Eijk LI, Wu G, Slungaard A, Mortari F, Griffioen AW, Mayo KH (2005). Platelet factor 4 and interleukin-8 CXC chemokine heterodimer formation modulates function at the quaternary structural level. J. Biol. Chem..

[16] Nguyen KTP, Druhan LJ, Avalos BR, Zhai L, Rauova L, Nesmelova IV, Dreau D (2020). CXCL12–CXCL4 heterodimerization prevents CXCL12-driven breast cancer cell migration. Cell Signal.

[17] Brown, A. J., Joseph, P. R., Sawant, K. V., & Rajarathnam, K. Chemokine CXCL7 Heterodimers: Structural Insights, CXCR2 Receptor Function, and Glycosaminoglycan Interactions. *Int. J. Mol. Sci*. **18**, 748-64 (2017). 10.3390/ijms18040748 PMC5412333 28368308

[18] Gouwy M, Struyf S, Mahieu F, Put W, Proost P, Van Damme J (2002). The unique property of the CC chemokine regakine-1 to synergize with other plasma-derived inflammatory mediators in neutrophil chemotaxis does not reside in its NH2-terminal structure. Mol. Pharmacol..

[19] Gouwy M, Struyf S, Catusse J, Proost P, Van Damme J (2004). Synergy between proinflammatory ligands of G protein-coupled receptors in neutrophil activation and migration. J. Leukoc. Biol..

[20] Struyf S, Gouwy M, Dillen C, Proost P, Opdenakker G, Van Damme J (2005). Chemokines synergize in the recruitment of circulating neutrophils into inflamed tissue. Eur. J. Immunol..

[21] Broxmeyer HE, Sherry B, Cooper S, Lu L, Maze R, Beckmann MP, Cerami A, Ralph P (1993). Comparative analysis of the human macrophage inflammatory protein family of cytokines (chemokines) on proliferation of human myeloid progenitor cells. Interacting effects involving suppression, synergistic suppression, and blocking of suppression. J. Immunol..

[22] Kuscher K, Danelon G, Paoletti S, Stefano L, Schiraldi M, Petkovic V, Locati M, Gerber BO, Uguccioni M (2009). Synergy-inducing chemokines enhance CCR2 ligand activities on monocytes. Eur. J. Immunol..

[23] Gouwy M, Struyf S, Noppen S, Schutyser E, Springael JY, Parmentier M, Proost P, Van Damme J (2008). Synergy between coproduced CC and CXC chemokines in monocyte chemotaxis through receptor-mediated events. Mol. Pharmacol..

[24] Zwijnenburg PJ, Polfliet MM, Florquin S, van den Berg TK, Dijkstra CD, van Deventer SJ, Roord JJ, van der Poll T, van Furth AM (2003). CXC-chemokines KC and macrophage inflammatory protein-2 (MIP-2) synergistically induce leukocyte recruitment to the central nervous system in rats. Immunol. Lett..

[25] Sebastiani S, Danelon G, Gerber B, Uguccioni M (2005). CCL22-induced responses are powerfully enhanced by synergy inducing chemokines via CCR4: Evidence for the involvement of first beta-strand of chemokine. Eur. J. Immunol..

[26] Grommes J, Alard JE, Drechsler M, Wantha S, Morgelin M, Kuebler WM, Jacobs M, von Hundelshausen P, Markart P, Wygrecka M, Preissner KT, Hackeng TM, Koenen RR, Weber C, Soehnlein O (2012). Disruption of platelet-derived chemokine heteromers prevents neutrophil extravasation in acute lung injury. Am. J. Respir. Crit. Care Med..

[27] Salanga CL, Handel TM (2011). Chemokine oligomerization and interactions with receptors and glycosaminoglycans: the role of structural dynamics in function. Exp. Cell Res..

[28] Miller, M. C., and Mayo, K. H. Chemokines from a structural perspective. *Int. J. Mol. Sci*. **18**. 2088-2104 (2017). 10.3390/ijms18102088 PMC5666770 28974038

[29] Chatterjee M, Huang Z, Zhang W, Jiang L, Hultenby K, Zhu L, Hu H, Nilsson GP, Li N (2011). Distinct platelet packaging, release, and surface expression of proangiogenic and antiangiogenic factors on different platelet stimuli. Blood.

[30] Veldkamp CT, Seibert C, Peterson FC, De la Cruz NB, Haugner JC, Basnet H, Sakmar TP, Volkman BF (2008). Structural basis of CXCR4 sulfotyrosine recognition by the chemokine SDF-1/CXCL12. Sci. Signal..

[31] Sepuru KM, Rajarathnam K (2021). Structural basis of a chemokine heterodimer binding to glycosaminoglycans. Biochem. J..

[32] Merkley N, Barber KR, Shaw GS (2005). Ubiquitin manipulation by an E2 conjugating enzyme using a novel covalent intermediate. J. Biol. Chem..

[33] Chen MJ, Mayo KH (1991). Human platelet factor 4 subunit association/dissociation thermodynamics and kinetics. Biochemistry-Us.

[34] Mayo KH, Roongta V, Ilyina E, Milius R, Barker S, Quinlan C, La Rosa G, Daly TJ (1995). NMR solution structure of the 32-kDa platelet factor 4 ELR-motif N-terminal chimera: a symmetric tetramer. Biochemistry-Us.

[35] Drury LJ, Ziarek JJ, Gravel S, Veldkamp CT, Takekoshi T, Hwang ST, Heveker N, Volkman BF, Dwinell MB (2011). Monomeric and dimeric CXCL12 inhibit metastasis through distinct CXCR4 interactions and signaling pathways. Proc. Natl. Acad. Sci. USA.

[36] Agten SM, Koenen RR, Ippel H, Eckardt V, von Hundelshausen P, Mayo KH, Weber C, Hackeng TM (2016). Probing functional heteromeric chemokine protein-protein interactions through conformation-assisted oxime ligation. Angew. Chem. Int. Ed. Engl..

[37] Bleul CC, Farzan M, Choe H, Parolin C, Clark-Lewis I, Sodroski J, Springer TA (1996). The lymphocyte chemoattractant SDF-1 is a ligand for LESTR/fusin and blocks HIV-1 entry. Nature.

[38] Oberlin E, Amara A, Bachelerie F, Bessia C, Virelizier JL, Arenzana-Seisdedos F, Schwartz O, Heard JM, Clark-Lewis I, Legler DF, Loetscher M, Baggiolini M, Moser B (1996). The CXC chemokine SDF-1 is the ligand for LESTR/fusin and prevents infection by T-cell-line-adapted HIV-1. Nature.

[39] Balabanian K, Lagane B, Infantino S, Chow KY, Harriague J, Moepps B, Arenzana-Seisdedos F, Thelen M, Bachelerie F (2005). The chemokine SDF-1/CXCL12 binds to and signals through the orphan receptor RDC1 in T lymphocytes. J. Biol. Chem..

[40] Burns JM, Summers BC, Wang Y, Melikian A, Berahovich R, Miao Z, Penfold ME, Sunshine MJ, Littman DR, Kuo CJ, Wei K, McMaster BE, Wright K, Howard MC, Schall TJ (2006). A novel chemokine receptor for SDF-1 and I-TAC involved in cell survival, cell adhesion, and tumor development. J. Exp. Med..

[41] Lasagni L, Francalanci M, Annunziato F, Lazzeri E, Giannini S, Cosmi L, Sagrinati C, Mazzinghi B, Orlando C, Maggi E, Marra F, Romagnani S, Serio M, Romagnani P (2003). An alternatively spliced variant of CXCR3 mediates the inhibition of endothelial cell growth induced by IP-10, Mig, and I-TAC, and acts as functional receptor for platelet factor 4. J. Exp. Med..

[42] Hattermann K, Holzenburg E, Hans F, Lucius R, Held-Feindt J, Mentlein R (2014). Effects of the chemokine CXCL12 and combined internalization of its receptors CXCR4 and CXCR7 in human MCF-7 breast cancer cells. Cell Tissue Res..

[43] Salazar N, Munoz D, Kallifatidis G, Singh RK, Jorda M, Lokeshwar BL (2014). The chemokine receptor CXCR7 interacts with EGFR to promote breast cancer cell proliferation. Mol. Cancer.

[44] Boldajipour B, Mahabaleshwar H, Kardash E, Reichman-Fried M, Blaser H, Minina S, Wilson D, Xu Q, Raz E (2008). Control of chemokine-guided cell migration by ligand sequestration. Cell.

[45] Naumann U, Cameroni E, Pruenster M, Mahabaleshwar H, Raz E, Zerwes HG, Rot A, Thelen M (2010). CXCR7 functions as a scavenger for CXCL12 and CXCL11. PLoS ONE.

[46] Levoye A, Balabanian K, Baleux F, Bachelerie F, Lagane B (2009). CXCR7 heterodimerizes with CXCR4 and regulates CXCL12-mediated G protein signaling. Blood.

[47] Holland JD, Kochetkova M, Akekawatchai C, Dottore M, Lopez A, McColl SR (2006). Differential functional activation of chemokine receptor CXCR4 is mediated by G proteins in breast cancer cells. Cancer Res..

[48] Weber C, Koenen RR (2006). Fine-tuning leukocyte responses: towards a chemokine 'interactome'. Trends Immunol..

[49] Wang X, Sharp JS, Handel TM, Prestegard JH (2013). Chemokine oligomerization in cell signaling and migration. Prog. Mol. Biol. Transl. Sci..

[50] Cojoc M, Peitzsch C, Trautmann F, Polishchuk L, Telegeev GD, Dubrovska A (2013). Emerging targets in cancer management: Role of the CXCL12/CXCR4 axis. Onco. Targets Ther..

[51] Ben-Baruch A (2008). Organ selectivity in metastasis: Regulation by chemokines and their receptors. Clin. Exp. Metastasis.

[52] Zlotnik A (2006). Involvement of chemokine receptors in organ-specific metastasis. Contrib. Microbiol..

[53] Peterson JE, Zurakowski D, Italiano JE, Michel LV, Connors S, Oenick M, D'Amato RJ, Klement GL, Folkman J (2012). VEGF, PF4 and PDGF are elevated in platelets of colorectal cancer patients. Angiogenesis.

[54] Dabrowska E, Przylipiak A, Zajkowska M, Piskor BM, Sidorkiewicz I, Szmitkowski M, Lawicki S (2020). Possible diagnostic application of CXCL12 and CXCR4 as tumor markers in breast cancer patients. Anticancer Res..

[55] Lehmann BD, Bauer JA, Chen X, Sanders ME, Chakravarthy AB, Shyr Y, Pietenpol JA (2011). Identification of human triple-negative breast cancer subtypes and preclinical models for selection of targeted therapies. J. Clin. Invest..

[56] Chavez KJ, Garimella SV, Lipkowitz S (2010). Triple negative breast cancer cell lines: one tool in the search for better treatment of triple negative breast cancer. Breast Dis..

[57] Wang Y, Li L, Wang H, Li J, Yang H (2018). Silencing TGIF suppresses migration, invasion and metastasis of MDAMB231 human breast cancer cells. Oncol. Rep..

[58] Dai X, Cheng H, Bai Z, Li J (2017). Breast cancer cell line classification and its relevance with breast tumor subtyping. J. Cancer.

[59] Crump MP, Gong JH, Loetscher P, Rajarathnam K, Amara A, Arenzana-Seisdedos F, Virelizier JL, Baggiolini M, Sykes BD, Clark-Lewis I (1997). Solution structure and basis for functional activity of stromal cell-derived factor-1; dissociation of CXCR4 activation from binding and inhibition of HIV-1. EMBO J..

[60] Gustavsson M, Wang L, van Gils N, Stephens BS, Zhang P, Schall TJ, Yang S, Abagyan R, Chance MR, Kufareva I, Handel TM (2017). Structural basis of ligand interaction with atypical chemokine receptor 3. Nat. Commun..

[61] Wescott MP, Kufareva I, Paes C, Goodman JR, Thaker Y, Puffer BA, Berdougo E, Rucker JB, Handel TM, Doranz BJ (2016). Signal transmission through the CXC chemokine receptor 4 (CXCR4) transmembrane helices. Proc. Natl. Acad. Sci. USA.

[62] Ziarek, J. J., Kleist, A. B., London, N., Raveh, B., Montpas, N., Bonneterre, J., St-Onge, G., DiCosmo-Ponticello, C. J., Koplinski, C. A., Roy, I., Stephens, B., Thelen, S., Veldkamp, C. T., Coffman, F. D., Cohen, M. C., Dwinell, M. B., Thelen, M., Peterson, F. C., Heveker, N., and Volkman, B. F. Structural basis for chemokine recognition by a G protein-coupled receptor and implications for receptor activation. *Sci. Signal*. **10**(471):eaah5756 (2017). 10.1126/scisignal.aah5756 PMC5648539 28325822

[63] Kufareva I, Stephens BS, Holden LG, Qin L, Zhao C, Kawamura T, Abagyan R, Handel TM (2014). Stoichiometry and geometry of the CXC chemokine receptor 4 complex with CXC ligand 12: Molecular modeling and experimental validation. Proc. Natl. Acad. Sci. USA.

[64] Korniejewska A, McKnight AJ, Johnson Z, Watson ML, Ward SG (2011). Expression and agonist responsiveness of CXCR3 variants in human T lymphocytes. Immunology.

[65] Mueller A, Meiser A, McDonagh EM, Fox JM, Petit SJ, Xanthou G, Williams TJ, Pease JE (2008). CXCL4-induced migration of activated T lymphocytes is mediated by the chemokine receptor CXCR3. J. Leukoc. Biol..

[66] Stephens, B. S., Ngo, T., Kufareva, I., and Handel, T. M. Functional anatomy of the full-length CXCR4-CXCL12 complex systematically dissected by quantitative model-guided mutagenesis. *Sci. Signal*. **13**(640):eaay5024 (2020). 10.1126/scisignal.aay5024 PMC7437921 32665413

