# Supplementary material for: A new obligate CXCL4–CXCL12 heterodimer for studying chemokine heterodimer activities and mechanisms
Source: Sci Rep. 2022 Oct 13;12:17204. doi: 10.1038/s41598-022-21651-0 (PMC9561612; doi:10.1038/s41598-022-21651-0)
Supplement: Supplementary file 1 — Supplementary Figures. [file 41598_2022_21651_MOESM1_ESM.pdf]

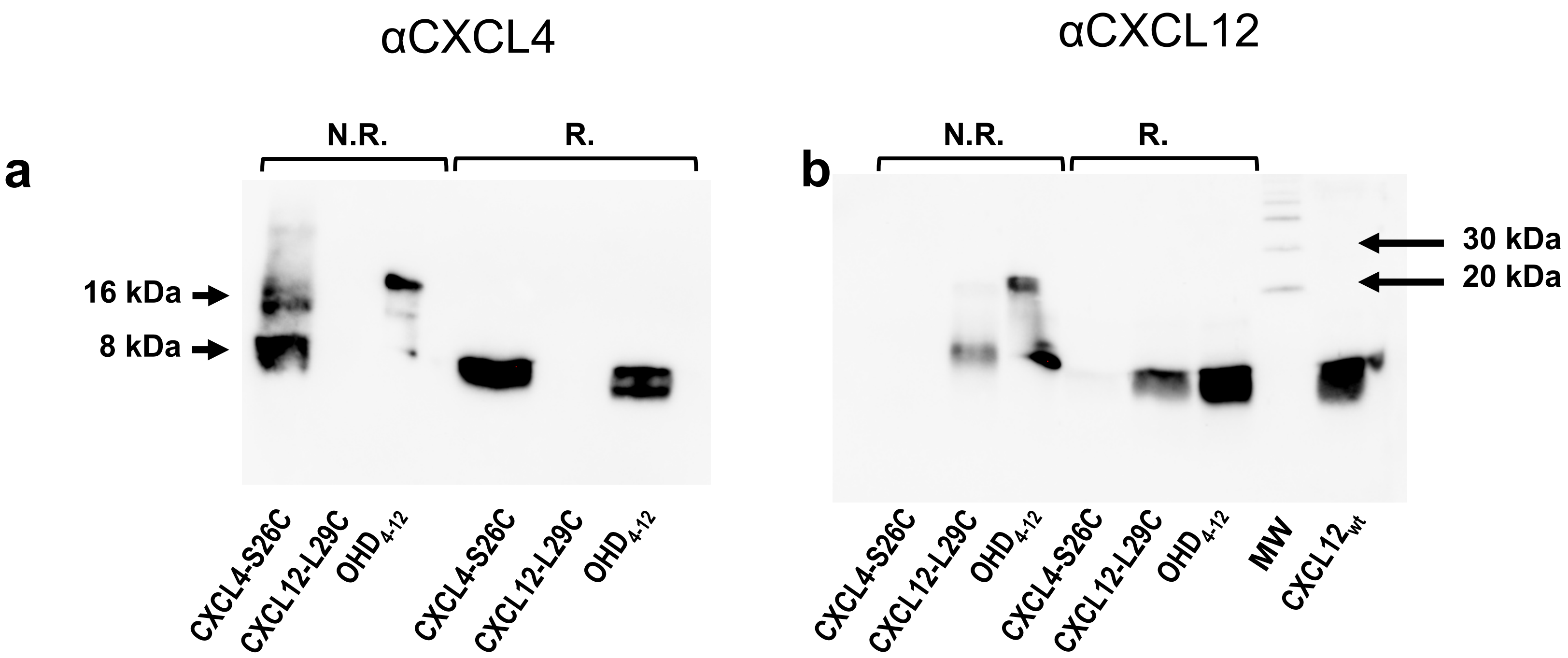

Supplementary Figure S1 (a-b). WB analysis in non-reduced (left bands, N.R.) and reduced (right bands, R.) conditions of $OHD_{4\text{-}12}$, CXCL4-S26C and CXCL12-L29C mutants, and $CXCL12_{wt}$ detected with anti-CXCL4 (αCXCL4, panel **a**) and anti-CXCL12 (αCXCL12, panel **b**) antibodies. Blots presented here are *in extenso* and are the basis for Figure 2b.

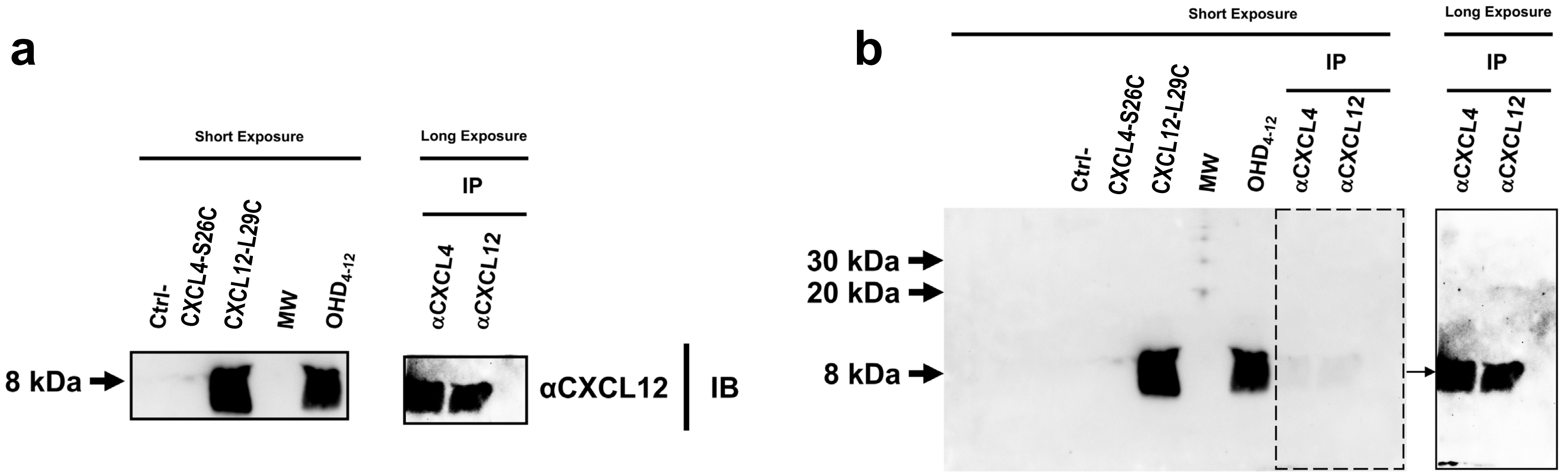

Supplementary Figure S2. Co-immunoprecipitation analysis. (a) $OHD_{4\text{-}12}$ and CXCL12-L29C mutant, but not CXCL4-S26C mutant, were detected by anti-CXCL12 (αCXCL12) immunoblotting (IP) (short exposure shown). Moreover, both immunoprecipitated (IP) $OHD_{4\text{-}12}$ fractions pulled with either anti-CXCL4 (αCXCL4) and αCXCL12 antibody were detected with αCXCL12 (long exposure shown). **(b)** The same data as presented in panel (**a**), but the full membrane (short exposure 2 min) (left) and long exposure (12 min) for IP wells following anti-CXCL12 (aCXCL12) IB (right) are shown. Bands are labeled as Ctrl- (i.e., FBS (10%) neg. ctrl), CXCL4-S26C, CXCL12-L29C, Protein MW (Magic Mark XP®), $OHD_{4\text{-}12}$, αCXCL4 (anti-CXCL4 IP fraction) and αCXCL12 (anti-CXCL12 IP fraction).

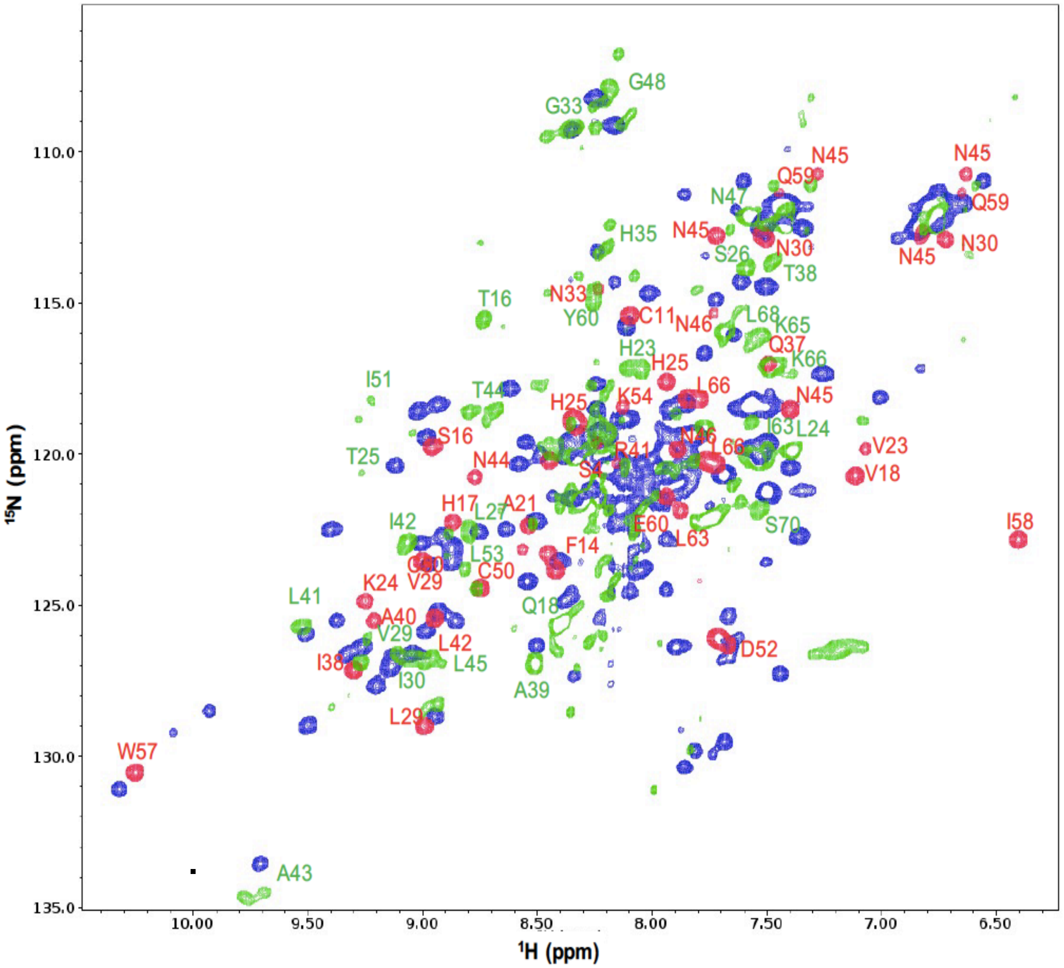

Supplementary Figure S3. The comparison of $OHD_{4\text{-}12}$, CXCL4, and CXCL12 15N-HSQC NMR spectra. Blue: the 15N-HSQC NMR spectrum of the uniformly 15N-labeled 68 μM $OHD_{4\text{-}12}$ in 90 % H2O/10 % D_2O at pH 6.9 in the presence of 20 mM NaCl, collected at 40 ^{o}C. Green: the 15N-HSQC NMR spectrum of the uniformly 15N-labeled 150 μM CXCL4 in 90 % H2O/10 % D_2O at pH 5.0 in the presence of 20 mM NaCl, collected at 40 ^{o}C. Red: the 15N-HSQC NMR spectrum of the uniformly 15N-labeled 51 μM CXCL12 in 20 MES buffer prepared with 90 % H2O/10 % D_2O at pH 6.8, collected at 25 ^{o}C. The difference in experimental conditions was due to different solubility properties of CXCL4 and CXCL12.

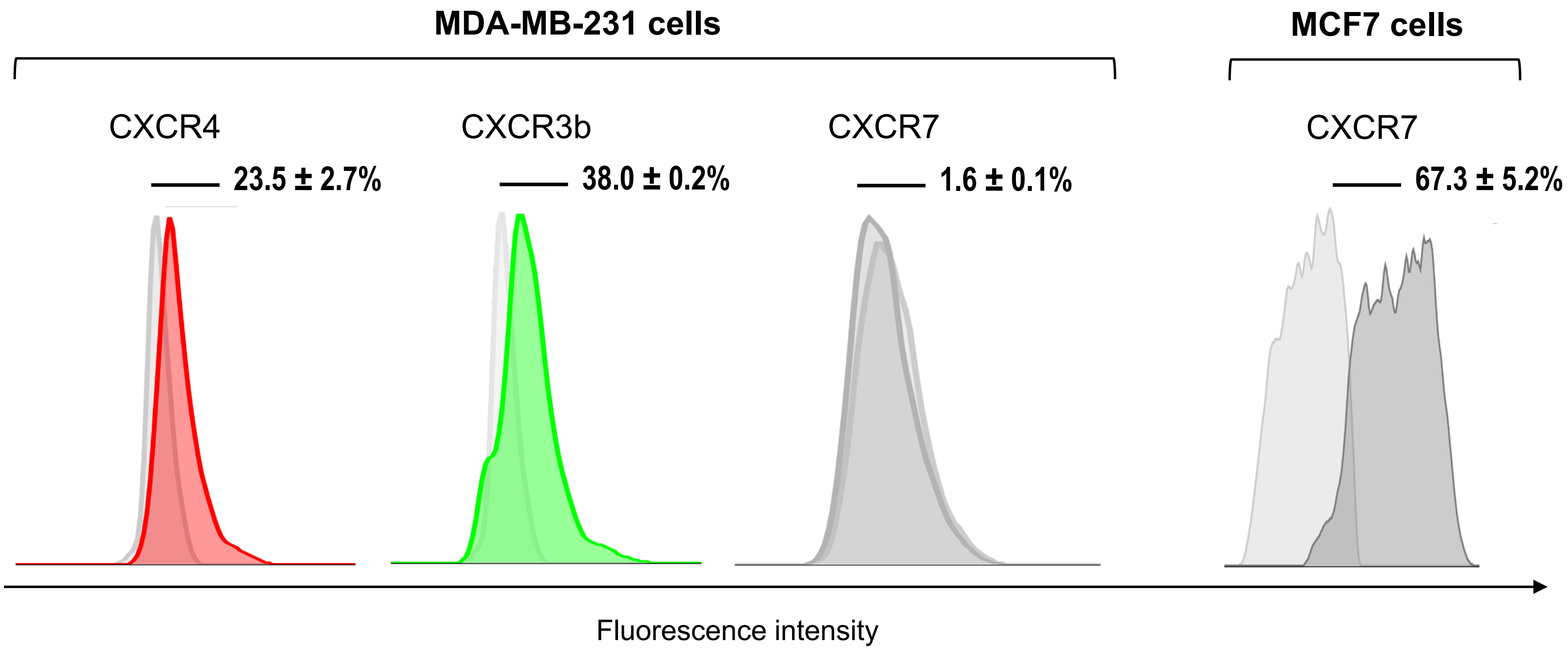

Supplementary Figure S4. The expression of CXCR4, CXCR3b, and CXCR7 receptors on MDA-MB-231 cells. Light grey represents isotype control, whereas red, green, and dark grey represent CXCR4, CXCR3b, and CXCR7, respectively. The expression of CXCR7 on MCF7 cells is shown for comparison. Flow-cytometry analyses were conducted as previously detailed (19).

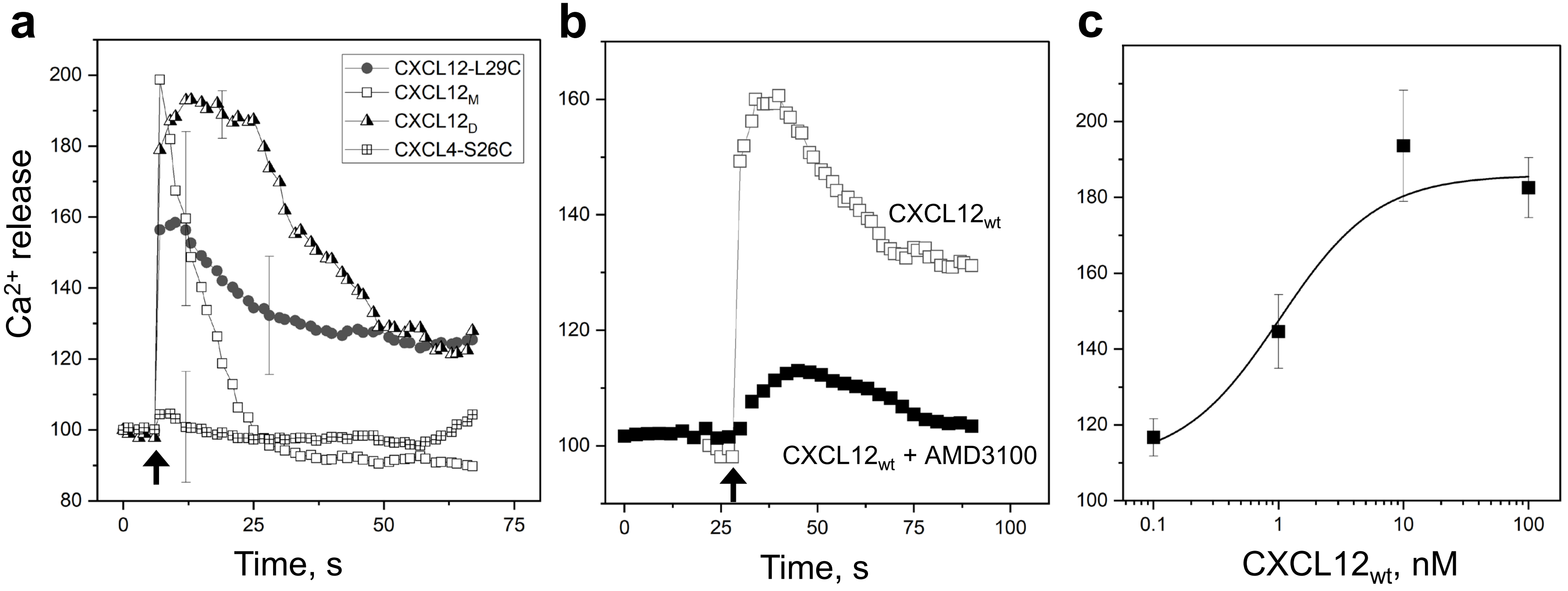

Supplementary Figure S5. The cytoplasmic Ca^{2+} release in MDA-MB-231 breast cancer cells. (a) The cytoplasmic Ca^{2+} release in MDA-MB-231 cells induced by 100 nM mutant CXCL12-L29C mutant, CXCL12 variants $CXCL12_M$ (obligate monomer) and $CXCL12_D$ (obligate dimer), and CXCL4-S26C mutant. The arrow indicates the time-point of chemokine addition. For each condition tested, standard deviations are exemplified. **(b)** The cytoplasmic Ca^{2+} release in MDA-MB-231 cells induced by 100 nM $CXCL12_{wt}$. The pre-incubation of cells with the CXCR4 antagonist AMD3100 (20 nM) inhibited the $CXCL12_{wt}$-induced intracellular Ca^{2+} release. The arrow indicates the time-point of chemokine addition. **(c)** The dose-response curve of intracellular Ca^{2+} release induced by $CXCL12_{wt}$. Solid line represents the best fit of experimental data using the logistic function. EC50 = 1.0 ± 0.5 nM.
